# Identity Integration in Adolescents With Features of Gender Dysphoria Compared to Adolescents in General Population

**DOI:** 10.3389/fpsyt.2022.848282

**Published:** 2022-06-09

**Authors:** Milla Karvonen, Kirstin Goth, Sami J. Eloranta, Riittakerttu Kaltiala

**Affiliations:** ^1^Department of Adolescent Psychiatry, Tampere University Hospital, Tampere, Finland; ^2^Department of Child and Adolescent Psychiatry, Psychiatric University Clinics (UPK) Basel, Basel, Switzerland; ^3^Faculty of Social Sciences, Tampere University, Tampere, Finland; ^4^Faculty of Medicine and Health Technology, Tampere University, Tampere, Finland; ^5^Vanha Vaasa Hospital, Vaasa, Finland

**Keywords:** gender dysphoria, identity development, gender identity, personality disorder, adolescence

## Abstract

Adolescence is an important period for identity formation and identity consolidation is one of the main developmental tasks. Gender identity is an essential aspect of identity but so far little is known about its development. Neither has the identity development of adolescents with features of gender dysphoria (GD) been extensively studied so far. However, adolescents with features of GD have been shown to present extensive psychiatric psychopathology and could therefore be assumed also to have more problems with identity development. We set out to compare the identity integration of adolescents with features of GD (*n* = 215; 186 natal females, 29 natal males) and adolescents from general population (*n* = 400; 244 females, 154 males and 2 who did not report their sex) using a culture-adapted Finnish version of an assessment tool for adolescents and young adults on identity in terms of personality functioning, the Assessment of Identity Development in Adolescence (AIDA). AIDA is a 58-item self-report questionnaire enabling dimensional differentiation between healthy and impaired identity development. The continuous AIDA total score (sum score) and its subscales were analyzed using MANOVA, and dichotomized *T*-scores differentiating identity development in impaired and healthy range using cross-tabulations with chi-square statistics. Adolescents with features of GD showed identity development similar to adolescents in general population. The slight differences seen in AIDA scores were in favor of the GD group. The proportion scoring to identity impairment was lowest among gender-referred adolescents assigned males at birth. Identity integration of the gender-referred adolescents was further compared to that of 77 adolescents in specialist level psychiatric outpatient treatment (67 females, 10 males). The adolescent psychiatric outpatients scored much higher toward impaired identity on all AIDA scales than did the adolescents with features of GD. These results suggest that features of GD are not associated with problems in identity development in adolescents at large. Adolescents with features of GD may have been required to process their identity more, thereby advancing further in their identity consolidation process than young people on average.

## Introduction

Identity and its favorable and unfavorable development play an essential role in both psychoanalytic/psychodynamic and socio-cognitive theories on the human mind. Identity is a broad concept encompassing both intrapsychic and interpersonal aspects and could therefore be roughly defined as a “unity of being” (1) or a “balance or interaction between that considered to be self and that considered to be other” (2). In general, identity can be said to allow one to move through life with a sense of continuity and purpose toward expressing one's goals and values.

According to many socio-cognitive theories, identity can be divided into two higher order domains “I” and “ME”, where the former emphasizes continuity, stable core, and emotional access, and the latter coherence, integrated whole, and cognitive access (1).

Within the psychodynamic tradition, Erikson (3) saw identity as a constant developmental process of ego growth. According to him, identity provides a sense of continuity within an individual and in contact with others (self-sameness) and also a means to differentiate between oneself and others (uniqueness), thereby enabling the individual to act independently from others. In this identity formation process, adolescence is a particularly important transitional period because the identifications of childhood are summed up, processed, and gradually replaced by a new form of identity. Additionally, the identities that form later in life are based on this adolescent identity to such an extent that they are perceptible as its variations (4). Hence, identity consolidation can be seen as a result of successful adolescent development.

Identity consolidation then involves, for example, experiencing oneself as consistent over time and situations, having stable values, long-term goals, and commitments [e.g., (5)]. Marcia et al. (6) elaborated on Erikson's concepts and developed a model including three identity statuses in addition to the achieved identity (that involved commitment after exploration): foreclosure, moratorium, and diffusion. In foreclosure, role and value commitments are made without exploration or struggle. Moratorium is a transitional phase and identity issues are put on hold due to occupational or other role commitments, which nevertheless signifies an active search for identity. Identity diffusion is more pathological and involves no commitments despite or without exploration, and can be seen, for example, in reoccurring changes in careers, values, and ideologies (5, 6). Identity diffusion can also be seen as a lack of integration of the concept of self and significant others [e.g., (7)].

The developmental process would ideally advance from initial childhood foreclosure or diffusion through moratorium to eventual identity achievement, which is challenged and reformed in connection with different life events (3, 8). However, it has been reported that large proportions of late adolescents and young adults do not complete the identity formation process, but nevertheless among those undergoing identity status transitions in late adolescence and young adulthood, progressive change is more than twice as likely than regressive change (9).

Identity can also be regarded as a core construct of personality (10). Hence, identity development can be seen as essential to personality development, with disturbances therein representing disturbances in personality development. Identity diffusion is considered to be one of the core elements of borderline personality organization (1, 7), and in the Diagnostic and Statistical Manual (DSM-5) (11), identity is classified as a central diagnostic criterion for personality disorders. In many countries, however, personality disorders are not diagnosed in adolescents. The life-span approach that promotes the diagnosing of children and adolescents and early intervention is gaining ground and is implemented in the new ICD-11 classification system (12) once it comes into effect.

Gender identity is one of the subareas of identity and refers to the person's own inner sense of their gender, which is distinct from the sex assigned to them at birth, which is based on their biological characteristics. This inner sense of gender does not necessarily align with the sex assigned at birth or the traditional expectations associated with it (13). Gender dysphoria (GD) refers to the distress experienced in relation to one's sex assigned at birth and the sex-discordant gender identity.

The process of gender identity consolidation is not known, but there are various theoretical perspectives on gender identity formation that view it as multi-dimensional, multi-determined, and open to change over time (2). For example, according to Bussey's model (14), aspects of individual, behavioral, and environmental level interact to co-create gender identity and likewise affect the change in gender identity over time. In this model, developmental change in gender identity is considered to be ongoing and continuous (14).

Gender identity in adolescence is nevertheless considered so stable that to relieve GD, international guidelines recommend interventions that modify physical sexual characteristics [e.g., (15)]. So far little research has been presented on GD in the context of identity development at large, or identity development among adolescents displaying GD. Adolescents with features of GD have been shown to present with excessive psychopathology (16–18) [along with, for example, excessive involvement in bullying (19)], and psychiatric disorders have been suggested to be associated with delayed or impaired identity development in general (20). Therefore, it could be assumed that they would also have problems in identity development, psychopathology either making them vulnerable to or representing impairment of identity development. Further research into the identity development of these adolescents would serve to determine their treatment needs as a group and thus help their treatment planning.

A recent Austrian study compared the presence of identity diffusion in adolescents with GD to population youth measured with the “Assessment of Identity Development in Adolescence” [AIDA; (21)]. AIDA is a self-report inventory to assess identity in terms of personality functioning and was specifically developed for adolescents and young adults (12–18 years old) to enable a dimensional differentiation between healthy and impaired identity development. Impaired identity development is assumed to be associated with a high risk of a current personality disorder, especially borderline personality disorder. Although identity diffusion was found in slightly over a third of the adolescents with GD, the overall results of this study suggested no pathological identity development for this group compared to same-aged population norms (21).

The tools used for the assessment of gender identity development are generally relatively one-dimensional, based on narrow concepts and do not yield information on identity development as a whole [for a recent assessment of the gender identity tools see (22)]. The assessment of overall identity development provides more reliable indications for proper individual treatment paths. AIDA is based on a broad and well-grounded theoretical framework and can offer an indicative building block in the detection of disturbances in identity development (1). It is not known whether the overall identity development of adolescents experiencing features of GD differs from the overall identity development of adolescents not experiencing these features. It seems probable that due to the significant psychopathology, adolescents with features of GD could also have more problems in their identity development, but on the other hand, they may have been required to process their identity more, which would yield them more favorable results than average.

The aim of this study was to assess the identity development of adolescents seeking treatment in a gender identity clinic due to GD and compare it to the identity development of adolescents in general population using the AIDA assessment tool. We set out to answer the following research questions:

1. Does the identity development of adolescents with features of GD requiring clinical assessment differ from that of same-aged adolescents in general population?
2. For how many of the adolescents with features of GD requiring clinical assessment are problems in identity development reaching levels indicating impairment in personality development?
3. How does the identity development of adolescents with features of GD requiring clinical assessment differ from that of adolescents requiring specialist level psychiatric care?

## Materials and Methods

This is a cross-sectional case-control study comparing 215 adolescents referred to a nationally centralized gender identity unit due to seeking gender affirming therapy and 400 population adolescents from four different schools participating in a study intended to collect data on three psychometric instruments recently translated into Finnish (23).

The data collection concerning the gender-referred adolescents comprised a retrospective chart review among adolescents with whom at least the initial assessments and interviews had been completed by the time of data collection in 2020. In Finland, mental health assessment of adolescents seeking medical gender affirming therapy is centralized to two university hospitals. A multi-disciplinary team comprising an adolescent psychiatrist, a psychologist, a social worker, and a psychiatric nurse carry out the assessments. The assessment of adolescents with features of GD takes place in an outpatient setting and comprises at minimum a review of their earlier medical and social welfare files and an initial assessment interview with the young person and their guardian(s). The AIDA is administered during the first visit and is the first structured measure used in the assessment process. Further assessments may not follow if more urgent treatment needs related to severe psychiatric disorders are evident at this stage; in such cases the young person is referred to appropriate care. If the gender identity assessment is continued, further free format and structured interviews and assessments are carried out, including the assessment of developmental history, adolescent development and personality, and specific gender identity related measures are also applied [see for example (18, 24)]. Gender affirming hormonal interventions can be initiated if there are no contraindications, such as severe psychiatric disorders that warrant treatment more urgently, and the young person has adequate caregiver support. Surgical treatments are possible after coming of age.

The gender-referred sample of the present study were 215 adolescents consecutively admitted to the gender identity unit for minors in Tampere University Hospital between 2016 and 2019. Of the gender-referred group, 15% were assigned males at birth, and 85% were assigned females at birth. Their mean (sd) age was 16.2 (1.3) years.

The participants from general population were 400 high school students from four different schools in three different cities in Finland. They responded anonymously using an Internet-based form during regular school hours in the spring term of 2019. Their parents received an electronic information letter before the students were recruited to participate, but active parental consent was not required. The students were informed by their teachers about the study and the voluntariness of participating. Students who did not want to participate were instructed to submit an empty form. Completed forms were taken as a consent to participate. The population adolescents had a mean (sd) age of 16.2 (1.5) years and 38.8% were males and 61.2% females; two students did not report their sex.

A clinical psychiatric comparison sample comprised 77 adolescents who were in specialist level outpatient adolescent psychiatric treatment in the Department of Adolescent Psychiatry in Tampere University Hospital between December 2020 and December 2022, were at least 15 years of age and consented to participate in a study project assessing suitability of selected psychometric instruments for a later psychotherapy outcome study. Consenting, otherwise unselected adolescent psychiatric outpatients filled anonymously in a set of psychometric instruments, including AIDA, and indicated their age and sex. Other background information was not collected. The adolescent psychiatric outpatient sample had a mean (sd) age of 16.6 (1.0) years, and 87% (67/77) were females. It is known that more than half of the outpatients in the study clinic have a primary diagnosis in the categories of severe mood disorders (F30–39) and anxiety disorders (F40–49) (25).

The register based clinical data collection was duly approved by the Ethics Committee of Tampere University Hospital, and the population study received ethics approval from Tampere University Ethics Committee.

### Measures

The “Assessment of Identity Development in Adolescence” (AIDA) is a self-report questionnaire enabling a dimensional differentiation between healthy and impaired identity development that is considered central in personality disorders, especially in borderline personality disorder (26). A detailed presentation of AIDA's theoretical underpinnings can be found at (27). AIDA contains 58 items with a 5-step answering format (0 = no to 4 = yes). All items are added up to obtain the total score representing Identity Diffusion. For descriptive purposes, the total score can be divided into two dimensions, Discontinuity and Incoherence. The continuity/discontinuity dimension has three subdimensions: consolidating perspectives and attributes (9 items; e.g., “*I could list a few things that I can do very well*.”), consolidating relationships and roles (11 items; e.g., “*I feel like I'm a valuable member of my family*.”), and consolidating emotional self-experience (7 items; e.g., “*Sometimes I have strong feelings without knowing where they come from*.”). The coherence/incoherence dimension also has three subdimensions: consistency in self-concepts (11 items; e.g., “*I often feel lost, as if I had no clear inner self*.”), autonomy and ego strength (12 items; e.g., “*If I am criticized or others see me failing, I feel really worthless and devastated*.”), and integrating cognitive self-experience (8 items; e.g., “*I am confused about what kind of person I really am*.”). This reflects the theoretical origins and complexity of the concept. All scores are coded toward pathology, that is, higher scores indicate pathology. Goth and Schmeck (26) reported very good scale reliabilities (0.94 for total score, 0.86 and 0.92 for main dimensions, and from 0.76 to 0.86 for subdimensions) for the original AIDA in German. Jung et al. (20) have demonstrated that AIDA can differentiate adolescents with personality disorder from the general population and also from adolescents with other types of psychiatric problems. Scores clearly above the average (T-scores above 60) denote probable risk for a current (borderline) personality disorder and an in-depth clinical investigation (e.g., with a clinical interview) is therefore recommended.

The culture-adapted version *AIDA Finnish* (28) was developed by the last author and her associates at the Universities of Tampere and Helsinki, Finland, in cooperation with the original authors. The original items were translated into Finnish with respect to cultural compatibility and were empirically tested in a pilot test with 77 adolescents. Based on the results, 10 items were slightly reformulated for the main test version without changing the targeted identity-related content in order to improve comprehensibility and reliability. The main test was performed in a combined sample of 400 adolescents from four schools and 129 adolescent psychiatric patients. The full sample consisted of 32.2% boys and 67.8% girls in the age range 12–21 years (mean = 16.2, SD = 1.4). To test the clinical validity of the Finnish version of AIDA, the sample was enlarged with 33 suicidal patients with diagnoses from the internalizing spectrum (20 with major depressive disorder). The AIDA Finnish scale reliabilities were good with Cronbach's alpha 0.96 on total, 0.90 and 0.95 on primary and 0.75–0.89 on subscale level. Exploratory factor analysis supported a one-factor solution speaking for a joint factor of “identity pathology”. The AIDA Finnish total score on Identity Diffusion differed at a highly significant level of *p* = 0.000 and with a large effect size of Cohen's *d* = 1.4 standard deviations between the adolescents of the school population (mean = 75.7, SD = 38.4) and the subsample of the 33 suicidal patients (mean = 126.0, SD = 30.7), mostly diagnosed with major depression based on K_SADS interview and a clinical classification conference after a thorough psychosocial evaluation of the young person and their family. This matches the results on clinical validity of the original Swiss-German AIDA version in patient groups with diagnoses from the internalizing spectrum (20).

In using self-report instruments, some information is always lost due to participants skipping individual questions or items of scales. AIDA Total score can be calculated if the number of missing items in the whole scale remains below 10%, and there is a maximum of 2 missing items per subscale. As per this rule, AIDA Total score and Discontinuity and Incoherence scores were successfully calculated for all participants in both population and clinical samples.

### Statistical Analyses

The data were analyzed using one-way multivariate analysis of variance (MANOVA) with SPSS 27. MANOVA is used to determine whether there are any differences between independent groups on more than one continuous dependent variable. Mean values of AIDA Total score and Discontinuity and Incoherence dimensions as well as of their respective subdimensions were compared between population males, population females, gender-referred adolescents assigned males at birth and gender-referred adolescents assigned females at birth. Within each sample, comparisons were made between sexes, and the gender-referred adolescents were also compared with population adolescents of the same and the opposite sex. Statistical differences between groups were assessed using Wilk's lambda, and effect sizes using partial eta squared (η2p). Statistical significance of differences in pairwise comparisons was analyzed using Tukey's *post-hoc* test, and due to multiple comparisons, cut-off for statistical significance was, using Bonferroni correction, set at *p* < 0.006. Next, we compared the proportions of general population males, general population females, and gender-referred adolescents assigned males and females at birth whose AIDA T-scores exceeded 60, which suggests impaired identity development, using cross-tabulations with chi-square statistics. Finally, AIDA Total score and Discontinuity and Incoherence dimensions as well as their respective subdimensions were compared between the gender-referred group and the general adolescent psychiatric outpatient sample.

## Results

Overall, there were statistically significant differences between the four groups on Identity diffusion and its subscales [*F*_(24, 1740.8)_ = 7.467, Wilks' Lambda = 0.753, *p* < 0.001]. AIDA Total score (=Identity diffusion), Discontinuity and Incoherence dimension and their subdimension scores are given in Table 1 for general population males, general population females, gender-referred assigned males at birth, and gender-referred assigned females at birth.

**Table 1:** AIDA total score, Discontinuity and Incoherence scale as well as their subscale scores by sex among population and gender-referred samples of Finnish adolescents [mean (sd)].

	**Population boys** ***n*** **=** **154**		**Population girls** ***n*** **=** **244**		**Gender-referred natal boys** ***n*** **=** **29**		**Gender-referred natal girls** ***n*** **=** **184**				
	**Mean**	**sd**	**Mean**	**sd**	**Mean**	**sd**	**Mean**	**sd**	***F*_(3;607)_**	***p* ^1^**	**Partial eta squared^**2**^**
**AIDA total score identity diffusion** ^**a**^	68.07	40.46	80.56	36.21	63.54	29.39	69.66	32.47	5.730	**0.001**	0.028
Discontinuity^b^	32.15	16.61	35.43	16.25	30.64	13.83	34.22	14.33	1.851	0.137	0.009
Consolid. perspectives^c^	13.18	6.045	13.95	5.85	11.17	5.58	12.51	5.36	3.451	0.016	0.017
Consolid. relationships^d^	10.47	7.70	10.69	6.39	12.36	5.79	13.32	5.66	7.454	**<0.001**	0.036
Consolid. emot. self-experience^e^	8.49	6.64	10.80	6.37	7.10	5.48	8.40	6.04	7.875	**<0.001**	0.037
Incoherence^f^	35.93	26.23	45.13	21.43	32.90	17.67	35.44	19.77	9.516	**<0.001**	0.045
Consistency in self^g^	13.68	9.50	16.24	8.86	13.33	8.45	13.29	7.80	5.067	**0.002**	0.024
Autonomy^h^	13.51	10.73	18.33	8.73	11.17	7.52	13.47	8.88	14.933	**<0.001**	0.069
Cogn self-exper^i^	8.75	7.18	10.56	5.85	8.38	4.74	8.68	5.25	4.821 (3,607)	**0.003**	0.023

*^1^p-values in this column indicate that overall, there are statistically significant differences between the groups on the dimension of the line; detailed findings of pairwise comparisons are explained in the text. P-values statistically significant after Bonferroni correction are highlighted in bold.*

*^2^Effect size η2p > 0.01 small, >0.06 medium, >0.14 large.*

*^a^Items per scale 58, range 0–232.*

*^b^Items per scale 27, range 0–108.*

*^c^Items per scale 9, range 0–36.*

*^d^Items per scale 11, range 0–44.*

*^e^Items per scale 7, range 0–28.*

*^f^Items per scale 31, range 0–124.*

*^g^Items per scale 11, range 0–44.*

*^h^Items per scale 12, range 0–48.*

*^i^Items per scale 8, range 0–32*.

The females in the population sample scored significantly higher than the population males on AIDA Identity Diffusion (*p* = 0.004), Discontinuity subdimension emotional self-experience (*p* = 0.002), primary dimension Incoherence (*p* < 0.001) and its subdimension autonomy and ego strength (*p* < 0.001). No significant differences between sexes were seen on any dimension or subdimension scores within the gender-referred group.

Gender-referred adolescents assigned males at birth did not differ statistically significantly from either general population males or females on AIDA Total score and its primary dimensions. Regarding subscales, they displayed a *lower* score on the autonomy and ego strength subscale of the primary dimension Incoherence than general population females (*p* = 0.001).

Gender-referred adolescents assigned females at birth did not differ from either population males or females on AIDA Total score and its primary dimension Discontinuity. They differed from general population females—but not from males—in displaying a lower score on primary dimension Incoherence (*p* < 0.001). Regarding subdimensions, gender-referred adolescents assigned females at birth differed from population females by displaying a *higher* score on the Discontinuity subdimension consolidating relationships and roles (*p* < 0.001) and *lower* scores on the Discontinuity subdimension consolidating emotional self-experience (*p* = 0.001) and on the Incoherence subdimensions consistency in self-concepts (*p* = 0.003) and autonomy and ego strength (*p* < 0.001), and borderline lower on the subdimension integrating cognitive self-experience (*p* = 0.007). They showed mostly subdimension scores comparable to those of general population males, differing only by scoring higher on discontinuity subdimension consolidating relationships and roles (*p* < 0.001; Table 1).

### Proportions of Population and Gender-Referred Adolescents Displaying Identity Pathology

In the general population sample, 14.2% of the scores reached a range suggesting impairment in identity development (*T*-score > 60) on AIDA total scale Identity Diffusion, whereas in the GD sample, the corresponding proportion was 9.8% (*p* = 0.07).

Among the general population adolescents, 15.8%, and in the GD group, 14.0%, of the scores reached a range suggesting impaired development on primary dimension Discontinuity (*p* = 0.32), and 15.0 and 9.3%, respectively, on Incoherence (*p* = 0.03).

The proportions reaching impaired range among males and females in the general population and birth-assigned males and females in the gender-referred sample are shown in Table 2. Pairwise comparisons between the groups revealed that the difference in proportions of those reaching impaired range in Identity diffusion was statistically significant only between gender-referred adolescents assigned males at birth and general population females (*p* = 0.05). For Discontinuity, none of the pairwise comparisons revealed statistically significant differences between the groups. For Incoherence, a statistically significant difference was in pairwise comparisons again seen only between gender-referred adolescents assigned males at birth vs. population females (*p* = 0.03).

**Table 2:** The proportion of those adolescents in each population/gender-referred group scoring within the pathological range (T-scores > 60) in Identity diffusion, Discontinuity and Incoherence [% (*n*/*N*)].

	**Population males**	**Population females**	**Gender-referred assigned males at birth**	**Gender-referred assigned females at birth**	***p***
Identity diffusion	11.0 (17/155)	16.4 (40/244)	3.4 (1/29)^a^	10.8 (20/185)	0.1
Discontinuity	13.5 (21/155)	17.2 (42/244)	6.9 (2/29)	15.1 (28/185)	0.5
Incoherence	11.0 (17/155)	17.6 (43/244)	3.4 (1/29)^b^	10.3 (19/185)	0.03

*^a^Difference to population females statistically significant at level p = 0.05.*

*^b^Difference to population females statistically significant at level p = 0.03*.

### Identity Development in Gender-Referred Adolescents Compared to Clinical Sample of Adolescent Psychiatric Patients

There were statistically significant differences between the gender-referred and the general adolescent psychiatric outpatient samples on Identity diffusion and its subscales [*F*_(8, 278)_ = 20.299, Wilks' Lambda = 0.631, *p* < 0.001, η2p = 0.37]. The gender-referred adolescents scored lower, indicating more favorable identity development, than the adolescent psychiatric outpatients on all AIDA scores (Table 3). Due to the very small number of males in the outpatient sample, this analysis could not be stratified by sex, but *post-hoc* analysis among only adolescents with female sex confirmed the systematic differences between the groups, all statistically significant at level *p* < 0.001.

**Table 3:** AIDA total score, Discontinuity and Incoherence scale as well as their subscale scores among gender-referred and adolescent psychiatric outpatient samples of Finnish adolescents [mean (sd)].

	**Gender-referred** ***n*** **=** **215**		**Outpatients** ***n*** **=** **77**				
	**Mean**	**sd**	**Mean**	**sd**	***F*_(1;287)_**	***p***	**Partial eta squared^**1**^**
**AIDA total score identity diffusion** ^**a**^	69.0	32.3	121.5	39.8	130.591	<0.001	0.314
Discontinuity^b^	33.8	14.5	54.2	17.6	96.729	<0.001	0.253
Consolid. perspectives^c^	12.5	5.4	18.6	6.4	64.315	<0.001	0.184
Consolid. relationships^d^	13.2	5.8	18.0	7.6	33.021	<0.001	0.104
Consolid. emot. self-experience^e^	8.2	6.0	17.6	6.6	125.314	<0.001	0.305
Incoherence^f^	35.1	19.7	67.8	23.7	136.040	<0.001	0.323
Consistency in self^g^	13.3	7.9	24.0	10.5	84.440	<0.001	0.229
Autonomy^h^	13.1	8.7	26.9	8.8	137.168	<0.001	0.325
Cogn self-exper^i^	8.6	5.2	16.7	6.8	109.796	<0.001	0.278

*^1^Effect size η2p > 0.01 small, >0.06 medium, >0.14 large.*

*^a^Items per scale 58, range 0–232.*

*^b^Items per scale 27, range 0–108.*

*^c^Items per scale 9, range 0–36.*

*^d^Items per scale 11, range 0–44.*

*^e^Items per scale 7, range 0–28.*

*^f^Items per scale 31, range 0–124.*

*^g^Items per scale 11, range 0–44.*

*^h^Items per scale 12, range 0–48.*

*^i^Items per scale 8, range 0–32*.

## Discussion

Finnish adolescents with features of GD in our study showed no marked pathological identity development as a group, which concurs with the results of the only study on the subject so far, conducted on Austrian youth [i.e., (21)]. The adolescents with features of GD did not differ from the population adolescents of either the same or the opposite sex regarding AIDA total score Identity integration and AIDA primary dimension Discontinuity. As to the primary dimension Incoherence, a difference in favor of gender-referred adolescents assigned females at birth in relation to population females was observed. Differences in subscale scores, when present, mostly suggested favorable development in the gender-referred adolescents in relation to population at large. Among the compared groups, the proportion scoring to identity impairment was lowest among gender-referred adolescents assigned males at birth.

The identity development of adolescents with features of GD has not yet been extensively studied and focusing on identity consolidation/identity diffusion at large is the unique contribution of the present study. Earlier research on identity in adolescents with features of GD has almost exclusively focused on the aspect of gender identity, ignoring the larger context of identity development at large.

In the only comparable study, by Haid-Stecher et al. (21), Austrian gender-referred adolescents scored to population norms on AIDA total score, primary dimensions and almost all their subdimensions. Our findings are slightly in favor of the gender-referred adolescents who on several scales displayed lower scores than their peers in population. The scores of the gender-referred adolescents in the present study also appear systematically lower, and thus more favorable, than the scores of the sample in the Austrian study (21). Adolescents presenting in gender identity services may differ between countries (29) for many reasons, such as differences in service systems and pathways to care as well as in societal acceptance of diversity.

Fewer of the adolescents with features of GD scored within the impaired range of Identity Diffusion compared to the young people in general population. This differs from the findings of Haid-Stecher et al. (21), where Identity Diffusion appeared likely in one third of the transgender adolescents, clearly exceeding the proportion in their population sample and also in our sample of gender-referred adolescents. Our finding suggests that for the vast majority of Finnish adolescents with features of GD, gender concerns are not associated with impaired identity development at large and, thus, do not indicate disturbances in their personality functioning.

There were no differences in the identity development between sexes among the adolescents with features of GD, whereas within the general population group females scored significantly higher than males on most comparisons (including total Identity Diffusion and primary dimension Incoherence). Higher scores suggest higher levels of impairment in general, however, the scores of the females did not reach levels anywhere near to identity pathology. It should be kept in mind that the clinical group of suicidal adolescents scored a mean 126.0 in AIDA total score (28).

It may be of interest to compare the scores of the adolescents with features of GD with those of the gender opposite to their birth-assigned sex in general population, and vice versa. In our study, the scores of the birth-assigned males with features of GD did not differ significantly from those of the population males or females (except for one subdimension in comparison to the population females). Meanwhile, the birth-assigned females in the gender-referred group differed on one primary dimension and some subdimensions from the general population females but not from the males. Hence, the identity development of the gender-referred females was slightly more mature than that of the population females and did not differ significantly from that of the population males. Additionally, analyses comparing proportions scoring to impaired range of identity development suggested least problems among gender-referred adolescents assigned males at birth.

This study suggests that the identity development trajectory may be slightly different among the adolescents with features of GD compared to that of adolescents on average, but in a positive way. The adolescents with features of GD may have been required to process their identity more and may thus have benefited from their possible identity crisis deriving from the experience of GD. This seems to be the case especially among the birth-assigned males with features of GD in our study, who seem to have been able to consolidate their identity most successfully, perhaps against many environmental odds, since feminine behavior in boys may be less readily tolerated than masculine behavior in girls (30, 31).

Excessive psychopathology has been reported in adolescents with GD (16–18), and severe psychopathology has been connected to poorer identity development (20). However, in our sample of gender-referred adolescents, identity development appeared normative and differed positively from the identity development of an adolescent psychiatric outpatient sample. However, more research is warranted on the role of psychopathology in identity integration among gender-referred samples.

### Limitations

Our study included 215 adolescents with features of GD, which yields quite a representative sample of this group in Finland. However, the number of birth-assigned males in the GD group was relatively low (*n* = 29), thus, further research with larger samples of birth-assigned males with GD is needed. The possibility should also be considered that those adolescents with features of GD who may simultaneously be suffering from more severe psychiatric comorbidities may not even have been referred to gender identity assessment and thus are not included in our sample. Additionally, the gender-referred adolescents in our sample were at the initial stage of the gender identity assessment process and were not (at least yet) diagnosed with Gender Dysphoria/ Transsexualism or receiving any gender affirming treatment.

Certain caution is warranted in interpreting the meaning of the finding of the slightly more favorable identity development in the gender-referred group as compared to the population one. Demographic and psychological variables, such as socioeconomic status and IQ, could differ between the groups and actually account for differences in identity development (32). Future research needs to proceed to exploring identity development taking into account such factors.

In addition to comparing identity development between gender-referred adolescents and same-aged peers in the general population, we were able to make comparisons between gender-referred adolescents and a sample of adolescents in specialist level psychiatric outpatient treatment. The gender-referred differed clearly from this clinical group, with the adolescent psychiatric outpatients displaying systematically significantly more impairment. Thus, gender-referred adolescents did not display general identity pathology as compared to population adolescents, and their identity development clearly differed from that of clinical adolescent psychiatric patients, the latter showing systematically more impairment. Future studies should further pursue to compare the identity development of gender-referred adolescents to different samples, also taking into account the possible psychopathology of the gender-referred adolescents. In the present data, no such background information was available. This is a limitation of the present study and warrants attention in future studies. It is known that adolescents presenting for gender identity assessment in the study clinic commonly present with severe psychiatric disorders and also differ from general population regarding sociodemographics, for example by living less commonly with both parents (18).

AIDA has been shown to have excellent clinical validity in detecting personality disorders, especially borderline personality disorder, and this has been shown in several languages (20, 26). Unfortunately, there was no Finnish borderline personality disorder sample to similarly test this clinical validity because diagnosing personality disorders before the age of 18 is not recommended in the ICD-10 (33), which is the diagnostic classification in use in Finland. However, the Finnish version is equivalent in all other result patterns to the original version of AIDA and has been developed step-by-step in cooperation with the original authors to ensure equivalence in the content. Moreover, a sample of Finnish suicidal adolescent psychiatric patients showed clearly elevated levels of impaired identity development. Persistent suicidality in adolescents may suggest borderline personality development (34), and, thus, findings among suicidal patients suggest that AIDA Finland likely differentiates personality pathology similarly to the original German version. Finnish version of the AIDA is, however, relatively recent, and research on its associations with dimensions of psychopathology in the general population is pending.

As concerns self-report materials at large, we cannot rule out attempts to present oneself overtly favorably or to exaggerate one's problems. Some of the gender-referred adolescents may have attempted to avoid discussion of possible mental health related needs, in hope of so prompting access to medical interventions. However, impression management when responding to AIDA is difficult as the underlying construct of identity development is complex and, therefore, it is not that easy to clearly understand the pathological reference of the full item set and manipulate the full result.

Different identity theories may use the same terminology to refer to different concepts and phenomena. In the work of Erikson, Marcia, and Kroger (3, 9), identity diffusion is an identity status characterized by lack of identity commitments and active identity work. In AIDA, the concept of identity diffusion refers, in line with Kernberg's theory of personality disorders, to a pathological identity development deemed a psychiatric syndrome underlying all severe personality disorders. Borderline personality organization in particular is characterized by identity diffusion manifesting in a non-integrated concept of the self and significant others (7, 20). In both these theoretical approaches, identity diffusion may manifest in a lack of commitment in a variety of life domains.

## Conclusion

In our study, the clinically referred adolescents with features of GD displayed similar or slightly more favorable identity development than did the Finnish young people in general. Adolescent birth-assigned males with features of GD presented with least impairment in their identity development. This does not suggest that transgender identification with feelings of GD would represent problems in identity development at large. The gender-referred adolescents with features of GD may have been required to process their identity more and, thus, may have benefited from the possible identity crisis related to GD. The potential progress in the identity development of these adolescents could be seen as their strength and taken into account when working with these adolescents, whether they progress further in their gender-affirming treatments or not.

## Data Availability Statement

The original contributions presented in the study are included in the article/supplementary material, further inquiries can be directed to the corresponding author.

## Ethics Statement

The studies involving human participants were reviewed and approved by Ethics Committee of Tampere University Hospital and Tampere University. Written informed consent from the participants' legal guardian/next of kin was not required to participate in this study in accordance with the national legislation and the institutional requirements.

## Author Contributions

RK analyzed the data. MK was responsible for drafting and revising the manuscript. All authors contributed to the design of the work, acquisition and interpretation of the data, revising the manuscript, and approving its submission to Frontiers in Psychiatry.

## Funding

This study has received financial support from Tampere University Hospital Research Grants (9X012 and 9AA022).

## Conflict of Interest

The authors declare that the research was conducted in the absence of any commercial or financial relationships that could be construed as a potential conflict of interest.

## Publisher's Note

All claims expressed in this article are solely those of the authors and do not necessarily represent those of their affiliated organizations, or those of the publisher, the editors and the reviewers. Any product that may be evaluated in this article, or claim that may be made by its manufacturer, is not guaranteed or endorsed by the publisher.
